# Radixin: Roles in the Nervous System and Beyond

**DOI:** 10.3390/biomedicines12102341

**Published:** 2024-10-15

**Authors:** Zhao Zhong Chong, Nizar Souayah

**Affiliations:** 1 Department of Neurology, New Jersey Medical School, Rutgers University, 185 S. Orange Ave, Newark, NJ 07103, USA; 2 Department of Neurology, New Jersey Medical School, Rutgers University, 90 Bergen Street DOC 8100, Newark, NJ 07101, USA

**Keywords:** radixin, ERM, phosphorylation, neurodegeneration, peripheral nerve injury, cancer

## Abstract

**Simple Summary:**

Radixin is a cytoskeletal-associated protein, a member of the ERM (ezrin, radixin, and moesin) protein family. Radixin plays important roles in cell shape, growth, and motility after activation by phosphorylation of its conserved threonine residues. Radixin functions as a relay in cell signaling pathways by binding to membrane proteins and transferring the cell signals into the cells. The pathogenic function of radixin has been found in central nervous system diseases, peripheral nerve injury, and cancers. We recently found significantly altered radixin in Schwann cells during elevated glucose, suggesting that it may be related to diabetes-induced nerve injury. As a result, the insight review into the roles of radixin and its associated cell signaling pathways may facilitate finding novel therapeutic targets for associated diseases.

**Abstract:**

Background: Radixin is an ERM family protein that includes radixin, moesin, and ezrin. The importance of ERM family proteins has been attracting more attention, and studies on the roles of ERM in biological function and the pathogenesis of some diseases are accumulating. In particular, we have found that radixin is the most dramatically changed ERM protein in elevated glucose-treated Schwann cells. Method: We systemically review the literature on ERM, radixin in focus, and update the roles of radixin in regulating cell morphology, interaction, and cell signaling pathways. The potential of radixin as a therapeutic target in neurodegenerative diseases and cancer was also discussed. Results: Radixin research has focused on its cell functions, activation, and pathogenic roles in some diseases. Radixin and other ERM proteins maintain cell shape, growth, and motility. In the nervous system, radixin has been shown to prevent neurodegeneration and axonal growth. The activation of radixin is through phosphorylation of its conserved threonine residues. Radixin functions in cell signaling pathways by binding to membrane proteins and relaying the cell signals into the cells. Deficiency of radixin has been involved in the pathogenic process of diseases in the central nervous system and diabetic peripheral nerve injury. Moreover, radixin also plays a role in cell growth and drug resistance in multiple cancers. The trials of therapeutic potential through radixin modulation have been accumulating. However, the exact mechanisms underlying the roles of radixin are far from clarification. Conclusions: Radixin plays various roles in cells and is involved in developing neurodegenerative diseases and many types of cancers. Therefore, radixin may be considered a potential target for developing therapeutic strategies for its related diseases. Further elucidation of the function and the cell signaling pathways that are linked to radixin may open the avenue to finding novel therapeutic strategies for diseases in the nervous system and other body systems.

## 1. Introduction

Radixin is one of the ERM family proteins, including ezrin, radixin, and moesin. ERM proteins, functioning as the link that bridges the actin cytoskeleton and membrane proteins, play very important roles in maintaining cell shape and motility through physically anchoring membrane proteins and assisting the signal transduction of post-translational processes [1]. ERMs also act as intracellular scaffolding proteins to relay the extracellular stimuli to the intracellular compartments of the cells [2]. In addition, ERMs have been demonstrated to regulate membrane dynamics and protrusion, cell adhesion, cell migration, and cell survival [2]. The broad cellular function of ERM implies that the deregulation of ERM holds potential roles in the development of diseases. In this article, we discussed the biological activity of ERM with a focus on radixin in a variety of diseases.

## 2. Activation of Radixin

### 2.1. Radixin Structure

**Figure 1:**
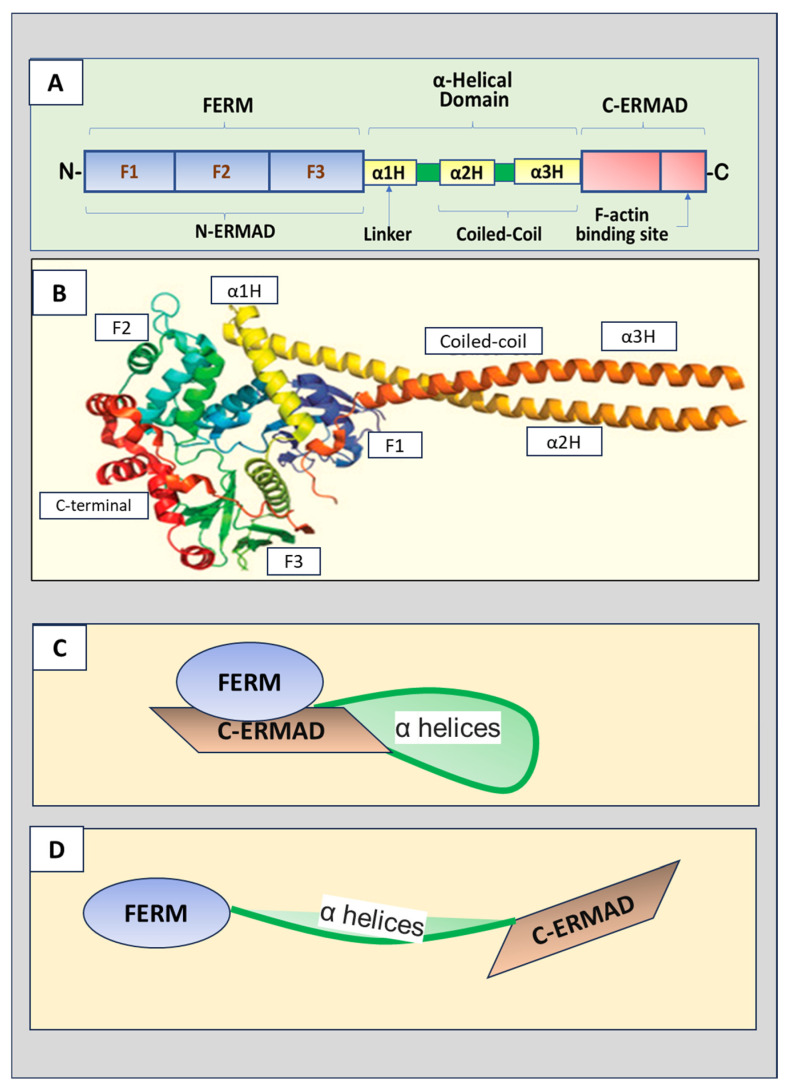
(**A**) Schematic ERM (ezrin, radixin, and moesin) protein domain structure. The N-terminus is the four-point-one, ezrin, radixin, moesin (FERM) domain that has F1, F2, and F3 subdomains. The FERM domain, also called N-terminal ERM association domain (N-ERMAD), is the site for ERM proteins to interact with the cell membrane. A central helical domain comprises three α helices, α1H, α2H, and α3H, which functions as a linker region connecting the FERM domain and an α-helical domain at the central portion of the protein. The α-helical domain can bind the FERM domain to facilitate the masking of both domains. The C-terminal end is the F-actin binding domain, also known as the C-terminal ERM-association domain (C-ERMAD), which has the ability to bind the FERM domain or F-actin. (**B**) The crystal structure of ERM proteins (reproduced from [3] and authorized by the publisher). (**C**) The inactive form of ERM proteins with C-ERMAD domain binding to and covering the FERM domain. (**D**) The active form of ERM proteins with the FERM domain released from the binding to the C-ERMAD domain.

The three ERM proteins possess similar structures, containing three major domains (Figure 1A,B). The amino terminus (N-terminus) is the four-point-one, ezrin, radixin, and moesin (FERM) domain that consists of F1, F2, and F3 subdomains (represented by A, B and C subdomains, respectively) [3]. The FERM domain, also named the N-terminal ERM-association domain (N-ERMAD), is the site for ERM proteins to interact with cell membranes. The FERM domain can bind to membranes, integral membrane proteins, and scaffolding proteins [4]. A central helical domain comprises three α helices, α1H, α2H, and α3H. α2H and α3H form a coiled-coil structure called the α-helical domain, while α1H acts as a linker region that connects the FERM domain and α-helical domain. The α-helical domain can bind to and mask the FERM domain. The carboxyl-terminal (C-terminal) end contains the F-actin binding domain, also known as the C-terminal ERM-association domain (C-ERMAD), which can bind the FERM domain and F-actin.

### 2.2. Radixin Activation

While performing its biological activities, ERM changes its conformation from inactive to active. The two confirmations are inactive closed form and active open form. The FERM domain of ERM proteins is shut in an inactive state due to the interaction of the N-terminal end with the C-terminal regions [2]. As shown in Figure 1C, in the inactive closed conformation, the C-ERMAD domain binds and covers both the F-actin and N-ERMAD (FERM domain), masking interaction sites of the FERM domain and F domain, leading to the loss of their binding ability to the membrane proteins, cytoskeletal protein, and other adaptor proteins [5]. Further studies indicated that the central α-helix-rich domain and linker regions also interact with F1 and F2 of the FERM domain, contributing to the masking of the binding sites [6].

To release the FERM domain from intermolecular binding, phosphorylation of conserved residue, threonine (Thr), in the FERM domain is required. Thr phosphorylation disrupts the binding between the FERM domain and the C-ERMAD region, relieving the FERM domain from the intramolecular association (Figure 1D). The phosphorylation of radixin has been demonstrated to disrupt the binding to the N-terminal domain to recover the binding ability of FERM without affecting the F-actin binding site. Phosphorylation of ezrin and moesin simultaneously unmasks both the F-actin and FERM binding sites. Ezrin is activated through phosphorylation of Thr567 at the C-ERMAD domain [7], leading to attenuating the affinity of the FERM domain to the C-ERMAD and reopening the binding sites of F-actin. The equivalent phosphorylation sites of radixin and moesin are Thr564 and Thr558, respectively [8]. However, phosphorylation of C-terminal Thr573 of radixin is required for both F-actin binding and improves protein stability [9].

### 2.3. Kinases and Radixin Phosphorylation

Many cellular kinases can phosphorylate the residues in C-ERMAD domain (Table 1). G-protein coupled receptor kinase 2 (GRK2) phosphorylates ezrin on Thr567, and is involved in membrane protrusion and motility in epithelial cells [10] and in G protein-coupled receptor-dependent cytoskeletal reorganization [11]. GRK2 regulates cell migration during wound recovery in epithelial cell monolayers, at least partly by phosphorylating radixin [12]. Nick interacting kinase (NIK)-induced phosphorylation of ezrin on Thr567 is necessary for lamellipodium extension induced by growth factors [13]. Lymphocyte-oriented-kinase (LOK) is a major ERM kinase in resting lymphocytes, and phosphorylation of ezrin regulates the cytoskeletal organization of lymphocytes [14]. Protein kinase C (PKC) phosphorylates ezrin to regulate osteosarcoma cell migration [15]. PKC-alpha has been shown to prefer ezrin as its target for phosphorylation [16], while PKC-theta prefers to phosphorylate moesin on Thr558 [17]. However, phosphorylation of ezrin by PKC-iota is essential for its normal distribution, and may be involved in the differentiation of intestinal epithelial cells [18].

In addition to the major regulatory threonine sites in the C-terminal region for the phosphorylation and activation of ezrin, other threonine positions or different residues have also been identified for ERM phosphorylation (Table 1). Cyclic-dependent kinase 5 (CDK5) phosphorylates ezrin on Thr235, which may also induce the association of ezrin with membranes and may be necessary for retinoblastoma tumor suppressor protein (pRb)-induced cell shape changes in senescent cells. In addition, CDK5-induced ezrin phosphorylation promotes Rho GDP dissociation inhibitor (Rho-GDI) to be separated from an ezrin/Rho-GDI complex, which plays an important role in cellular morphology in the process of senescence [21]. Phosphorylation of Thr235 works with phosphorylation of Thr576 for the full activation of ezrin, which is involved in the induction of osteosarcoma cell morphology changes during senescence [21]. In addition, Src kinases (SK) and the intrinsic Tyr kinase (ITK) can phosphorylate ezrin on Tyr145, 353, and 477. Phosphorylation of ezrin on Tyr145 and 477 has been involved in cell adhesion and migration, whereas Tyr353 phosphorylation contributes to the reorganization of the actin cytoskeleton and tetraspanin CD81- and B-cell receptor (BCR)-mediated activation of B cells [31,32].

Regarding moesin, Rho-associated kinase (RhoK)-induced phosphorylation of moesin at Thr558 regulates the formation of microvilli structures [23]. RhoK also mediates cortical neuron growth, morphological changes, and motility through phosphorylation of ERM proteins [25]. Cell division control protein 4 (Cdc42) phosphorylates moesin, which is involved in filopodia formation [28]. Phosphorylation of moesin by glutamate in hippocampal neuronal cells is dependent on RhoA and Rho kinase [24]. Activation of Akt, also named protein kinase B, induced neurite outgrowth requires phosphorylation of moesin on Thr558 in PC12 cells [26]. Rho-associated coiled coil-containing protein kinase (ROCK) phosphorylates T567 of ezrin and T558 of moesin to regulate Fas-mediated Jurkat cell apoptosis [27].

Akt promotes the outgrowth of neurite by enhancing the interaction of radixin with F-actin through phosphorylating radixin at Thr573 [9], since inhibition of Akt-induced radixin phosphorylation reduces the affinity of radixin and F-actin binding, decreases the outgrowth of neurites, and reduces the formation of the growth cone. Moreover, radixin phosphorylation by Akt on the Thr573 residue also results in inhibiting ubiquitin-dependent proteasomal degradation of radixin, improving radixin protein stability and increasing neurite outgrowth. In contrast, suppression of Akt-dependent phosphorylation of radixin results in reduced radixin levels and decreased neurite outgrowth and growth cone formation [9].

Thrombin has been demonstrated to promote the phosphorylation of radixin at Thr564, ezrin at Thr567, and moesin at Thr558 in a PKC-dependent manner to regulate endothelial hyperpermeability [20]. In addition, guanine nucleotide-binding protein Galpha1 (G13) protein, one of the heterotrimeric G proteins, has also been associated with radixin activation [34].

### 2.4. Radixin Binding to the Cell Membrane

The phosphorylation of ERM and the subsequent openness of its domains results in its binding to phosphoinositides (4,5)P_2_ (PIP_2_) by its FERM domain. PIP_2_ could mediate the recruitment of ERMs to the plasma membrane and induce the translocation of plasma proteins. There are three lysine-rich consensus sites known to bind phosphoinositides on the FERM domain; PIP_2_ can bind to ERMs through the sites of Lys63-Lys68 on the F1 subdomain and the clusters of Lys253-Lys254 and Lys262-Lys263 on the subdomain F3 of the FERM domain [35,36]. Recently, the binding of FERM domains to PIP_2_ in lipid bilayers has significantly improved the binding stability of FERM to the membrane [37].

## 3. Expression of Radixin

Radixin is expressed ubiquitously in cells. Radixin was originally found in adherence junctions in rat liver. The expression of ERM proteins was found at cell surface structures, including apical microvilli, ruffling membranes, filopodia, uropods, retraction fibers, the cleavage furrow of dividing cells, and adjacent sites of actin filaments and the plasma membrane [38,39].

Although ERM proteins are present in most cells, their expression levels are in cell- and organ-specific manners. For example, nature-killer cells expressed all three ERM proteins; however, no expression of radixin was found in nature-killer cell-deprived peripheral blood leukocytes and T lymphocytes [40].

In the nervous system cells, myelinating Schwann cells express all three ERM proteins [41]. In the brain, radixin expression has been found in the cerebral cortex, striatum, cerebellum, thalamus, hippocampus, and the olfactory bulb [42]. Cellularly, radixin is expressed in oligodendrocyte transcription factor 2 (OTF-2)-positive cells, neural progenitor cells, and activated microglia. Interneurons and primary cultured astrocytes have also been demonstrated to express radixin. The expression of radixin neural progenitor cells in the subventricular zone (SVZ) and the rostral migratory stream (RMS) may implicate its role in neuroregeneration [43,44].

Radixin is also highly expressed in liver cancer cells and human gastric carcinoma cells [45].

## 4. The Role of Radixin and Cell Signaling Pathways

**Figure 2:**
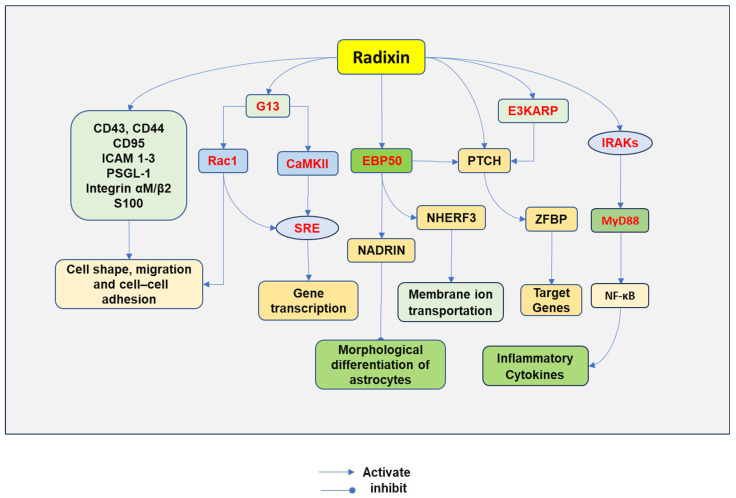
Radixin-induced cell signaling pathways. Radixin can bind directly to the cytoplasmic tails of many membrane proteins, including CD44, CD43, CD95, intracellular adhesion molecule 1-3 (ICAM 1-3), L-selectin, P-selectin glycoprotein ligand-1 (PSGL-1), integrin alpha M/beta2, calcium-binding EF-hand-like S100 protein, regulating cell shape, migration, and other cell interacting processes. Radixin can also interact with proteins through the adaptor protein ERM-binding phosphoprotein 50 (EBP50) and NHE3 kinase A regulatory protein (E3KARP). Na^+^/H^+^ exchange regulatory cofactor (NHERF3) can bind to EBP50 and undergo a conformational change. Patched protein (PTCH), a Hedgehog receptor, can bind to EBP50 or directly to radixin. The binding of Hedgehog to PTCH induces zinc finger-domain-bearing protein (ZFBP) and subsequent expression of its target genes. Neuron-associated developmentally regulated protein (NADRIN) interacts with EBP50, leading to its inactivation of and morphological differentiation of astrocytes. Radixin can regulate Galpha13 (G13)-mediated signaling pathways. Radixin mediates serum response element (SRE)-dependent gene transcription through activation of Ras-related C3 botulinum toxin substrate 1 (Rac1) and calmodulin-dependent protein kinase (CaMKII). Radixin-mediated Rac1 can also regulate cell shape, migration, and cell–cell adhesion. Radixin may be involved in the lipopolysaccharide-Notinduced release of proinflammatory cytokines through nuclear factor (NF)-κB by recruiting interleukin-1 receptor-associated kinases (IRAKs)/myeloid differentiation primary response 88 (MYD88) followed by activation of NF-κB, leading to an increase in gene expression of inflammatory cytokines.

ERM proteins play a crucial role in cytoskeletal regulation through protein interactions. Inactive ERM proteins are mainly distributed in the cytoplasm of cells, while activated ERMs attach to the membrane (Figure 2).

### 4.1. Relaying the Cell Signaling

ERM functions not only as a bridge between the membrane and the cell cytoskeleton, but is also involved in cell signaling pathways. ERM regulates plasma membrane localization and functional activity. ERM initially anchors transmembrane proteins on the plasma membrane and facilitates multiple protein–protein interactions. The FERM domain of ERM proteins can bind directly to the cytoplasmic tails of many membrane proteins, including CD44, CD43, CD95, intracellular adhesion molecule 1-3 (ICAM 1-3), L-selectin, P-selectin glycoprotein ligand-1 (PSGL-1) [46], and integrin alpha M/beta2 [47]. In addition, ezrin has also been shown to bind to calcium-binding EF-hand-like S100 protein by its FERM domain [48]. Protein A-kinase is also an interacting protein, in which the α-helical linker region of the ERM protein binds to its regulatory subunit, while F-actin binds its tail [49].

### 4.2. Binging through Adaptor Proteins

ERM can also indirectly interact with different membrane proteins through adaptor proteins. There exist some adaptor proteins for ERMs, including ERM-binding phosphoprotein 50 (EBP50), membrane-type 1 matrix metalloproteinase (MT1-MMP) [50], and NHE3 kinase A regulatory protein (E3KARP) [51]. EBP50 and E3KARP have two postsynaptic density 95/disks large/zona occludens-1 (PDZ) domains and an ERM-binding domain. EBP50 and E3KARP have been found to bind multiple different proteins [52]. Na^+^/H^+^ exchange regulatory cofactor (NHERF3), also named PDZK, has four PDZ domains that can bind to EBP50 and undergo a conformational change [53]. The expression of NHERF3 is found in the brush border of epithelial cells of the renal proximal tubule and the gastrointestinal tract, functioning to regulate membrane ion transportation [54].

### 4.3. Regulating PTCH Cell Signaling

Patched protein (PTCH) emerges as a membrane receptor that is regulated by ERM proteins. PTCH is a conserved 12-pass transmembrane protein receptor that negatively regulate the Hedgehog signaling pathway. Hedgehog binds to its receptor PTCH, thereby inducing zinc finger-domain-bearing protein (ZFBP) and subsequent expression of its target genes [55].

PTCH interacts with the FERM domain directly or indirectly through EBP50 and E3KARP [56]. The binding of these proteins may function to link PTCH to the cell cytoskeleton and promote the formation of a signaling complex with its ligands [8].

### 4.4. Mediating the Differentiation of Astrocytes

ERM has been associated with neuron-associated developmentally regulated protein (NADRIN). The Rho subfamily of small GTPases regulates cell skeleton control. The induced expression of NADRIN promoted the morphological differentiation of cultured astrocytes into stellate cells. Further study indicated that NADRIN interacted with EBP50 via its C-terminal PDZ-binding motif to form a complex with ERM, leading to its inactivation and morphological differentiation of astrocytes [57].

### 4.5. Regulating Inflammatory Cascade

ERM is also involved in lipopolysaccharide (LPS)-induced release of proinflammatory cytokines through activating nuclear factor (NF)-κB. LPS-induced ezrin phosphorylation is dependent on RhoA/ROCK, leading to ezrin translocation to the cell membrane where it recruits interleukin-1 receptor-associated kinase 1 (IRAK-1) and myeloid differentiation primary response 88 (MYD88), followed by activation of NF-κB and a subsequent increase in gene expression of inflammatory cytokines. The inhibition of ezrin reduced LPS-induced production of tumor necrosis factor-α (TNF-α), interleukin-1β (IL-1β), and high mobility group box 1 protein (HMGB1) [58].

Moesin has also been associated with the release of inflammatory cytokines. LPS can phosphorylate moesin and induce the binding of moesin to TLR4, which activates MyD88. MyD88 then activates interleukin-1 receptor-associated kinase (IRAK) through their respective death domains. IRAK is autophosphorylated, leading to its dissociation from MyD88. IRAK interacts with TNF receptor-activated factor 6 (TRAF6). Active TRAF6 then phosphorylates mitogen-activated protein kinase kinase 1 (MEKK-1 or MAP kinase kinase (MKK)3/6, MKK4, MAP kinase/ERK kinase (MEK), and B-inducing kinase (NIK). Both NIK and MEKK-1 activate IKK, which phosphorylates IκBα, leading to the degradation of IκBα and freeing of NF-κB from the cytoplasmic binding. NF-κB is then translocated from the cytoplasm to the nucleus, where it promotes the transcription of genes of inflammatory cytokines [59].

### 4.6. Regulating G13-Induced Cell Signaling

Radixin can regulate G13-mediated cell signaling transduction. The C-terminal domain of radixin regulates serum response element (SRE)-induced gene transcription through activation of Ras-related C3 botulinum toxin substrate 1 (Rac1) and calmodulin-dependent protein kinase (CaMK) II, while the N-terminal domain potentiates the activation of SRE induced by G13. Both radixin and active G13 can activate Ca^2+^/CaMKII. Active radixin stimulates Rac1, phosphorylates CaMKII, and induces SRE-dependent gene transcription. Small interference RNA against radixin suppressed SRE-dependent gene transcription, and active G13-induced CaMKII phosphorylation [60].

Radixin mediates Rac1 to regulate cell morphology, migration, and cell interaction. The depletion of radixin affects cell morphology, migration, and cell adhesion [61]. Interestingly, the interaction between Rac1 and radixin plays important roles in cytoskeletal remodeling, cell adhesion, and cell motility [61]. Downregulation of Rac1 expression decreased radixin knockdown-induced cell area increase. In contrast, constitutively active Rac1 resulted in cell spreading and increased expression of N-cadherin at cell–cell contacts [61].

## 5. The Roles of Radixin in the Nervous System

### 5.1. Promoting the Growth of Neurons

**Figure 3:**
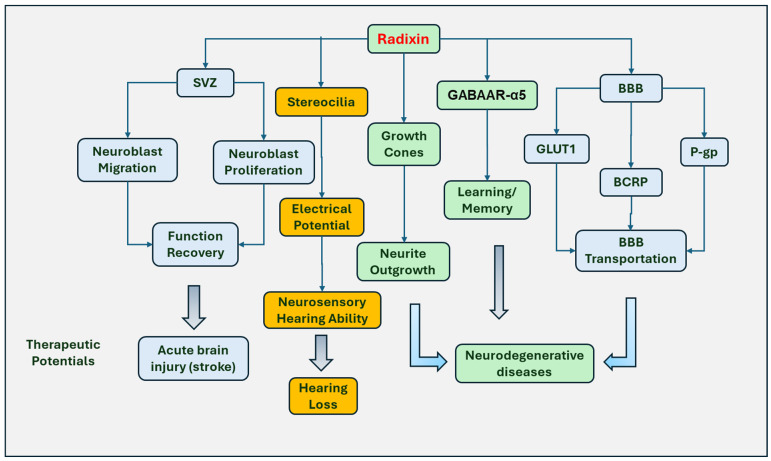
The roles of radixin in the nervous system. Radixin promotes neural progenitor cell migration and neuroblast proliferation in the subventricular zone (SVZ) and the rostral migratory stream, possibly promoting functional recovery after brain injury. Radixin also plays a role in neurite formation and the development of neuronal polarity *via* regulating growth cone development and maintenance. Radixin is expressed in stereocilia of the inner ear sensory cells and is necessary for the conversion of sound into electrical signals at an acoustic rate. Moreover, radixin is necessary for γ-Aminobutyric acid type A (GABAA) receptor alpha5 subunit (GABAAR-α5) to anchor at the actin cytoskeleton to mediate tonic inhibition and hippocampal-dependent short-term memory. Furthermore, radixin has been shown to function to maintain the plasma membrane localization and transport activities of P-glycoprotein (P-gp), breast cancer resistance protein (BCRP), and glucose transporter 1 (GLUT1) proteins through the blood–brain barrier (BBB) illustrated in an *in vitro* model, which may facilitate the access of therapeutic drugs into the brain. Accordingly, targeting radixin has the potential for the treatment of stroke, neurodegenerative diseases, and hearing loss.

Radixin promotes neural progenitor cell migration and neurite outgrowth (Figure 3). Neuroblasts, neuronal progenitor cells in the neurogenic SVZ, can migrate to the olfactory bulb in the rodent brain through RMS. The system plays an critical role in functional recovery during brain injury, which has been shown to induce neuroblast migration to the damaged tissue areas after stroke [62] and rodent cerebral ischemia [63,64]. Radixin is highly expressed in neuroblasts of the adult RMS and SVZ [42]. The involvement of radixin in neuroblast migration was illustrated when a radixin inhibitor was applied to the neuroblast cultures. The results indicated that radixin functional inhibitor DX52-1 reduced neuroblast migration without affecting glial migration. Interestingly, radixin inhibition was shown to decrease the proliferation of neuroblasts only without inhibition of other cell proliferation in the RMS [42].

Radixin has been associated with axonal outgrowth, morphological rearrangement, and cell migration. Radixin and moesin were reported to promote the growth of neurites and the formation of neuronal polarity *via* regulating growth cone development and maintenance. The study demonstrated that double suppression of radixin and moesin reduced the growth cone size and caused disorganization of actin filaments, resulting in the formation of short neurites and impaired development of an axon-like neurite [65]. Radixin is also involved in the stability of lamellipodia during nerve growth cone motility. In chick dorsal root ganglion growth cones, inactivation of radixin causes a 30% reduction of lamellipodial area [66]. Activation of ERM by RhoK plays a role in growth, morphologic change, and motility regulation of cortical neurons *in vitro* [25]. Inhibition of RhoK significantly prevented neurite outgrowth accompanied by decreased ERM phosphorylation [25]. Leucine-rich repeat kinase-2 (LRRK2) phosphorylates moesin at Thr558, ezrin at Thr567, and radixin at Thr564 [29]. G2019SA is a Parkinson’s disease-related substitution in the kinase domain of LRRK2. LRRK2, with this substitution, perturbs the homeostasis of active ERM and F-actin in sprouting neurites. In cultured neurons derived from LRRK2 G2019S transgenic mice, a significantly increased number of active ERM-positive and F-actin-enriched filopodia was observed, which correlates with the retardation of neurite outgrowth [30].

Radixin is also involved in the regenerative process. ERM acts as a binding partner of the L1 cell adhesion molecule (L1CAM). The binding can regulate the regeneration response after injury [67]. In neuronal culture from the hippocampus and cerebral cortex, ERM proteins were accumulated in the growth cones of sprouting neuronal processes after neurite transection. Significantly longer regenerative neurites were observed in the cultures with L1CAM as substrate compared to cultures with poly-L-lysine as substrate [68] (Figure 3).

### 5.2. Regulating Hearing Function

Radixin is an important component for maintaining hearing function. Radixin expresses on the sensory cell stereocilia of the inner ears and modulates the function of the stereocilia. Radixin seems to be necessary for the conversion of sound into electrical signals, since inhibition of radixin resulted in decreased sound-evoked electrical potentials on stereocilia [69]. In addition, the null allele of the *radixin* gene resulted in the degeneration of inner ear hair and hearing loss in mice [70], while two mutant alleles of radixin (D578N and A469fsX487) have been linked to neurosensory hearing loss [71]. In contrast to the homozygous depletion of radixin that causes stereocilial degeneration that leads to hearing loss, monoallelic loss of radixin promotes the startle reflex induced by acoustic stimulation with increasing intensities [72].

### 5.3. Involved in Learning and Memory Processing

Radixin is an anchor for important signaling proteins, functioning in memory and the learning process. γ-Aminobutyric acid type A (GABAA) receptor alpha5 subunit (GABAAR-α5) is located in the extrasynaptic system *via* radixin-mediated anchorage and is thought to mediate tonic inhibition. Radixin was shown to be required for GABAAR-α5 binding since loss of radixin or F-actin binding motif impairs GABAAR-α5 cluster formation [73]. Phosphorylation of radixin resulted in uncoupling of GABAAR-α5 from the extrasynaptic anchor, thereby increasing synaptic receptor numbers. In contrast, the inactivation of radixin impairs the stability of GABAAR-α5, which dissociated from the synaptic exterior and thereby increases its spatial distribution in soma [74]. Radixin depletion impairs short-term memory and impairs reversal learning in mice. The data suggest that radixin mediates synaptic GABAAR density and regulates reversal learning and short-term memory [44].

### 5.4. Regulating Transport through the BBB

Moreover, radixin also functions to maintain the plasma membrane localization and transportation of P-glycoprotein (P-gp), glucose transporter 1 (GLUT1), and breast cancer resistance protein (BCRP) in a blood-brain barrier (BBB) model. However, radixin has a different function from ezrin and moesin in regulating the transporters on the plasma membrane and their efflux activities. Radixin knockout reduced the membrane expression of all three transporters. The knockdown of ezrin or moesin reduced the expression of BCRP and GLUT1 on the plasma membrane *in vitro* BBB. However, these effects are not consistent with their effects on efflux activity. Moesin knockdown most potently decreased efflux activity of P-gp and BCRP, whereas knockdown of all three ERM proteins similarly reduced GLUT1 influx activity [19]. This role may affect the access of drugs into the central nervous system, which may facilitate the access of therapeutic drugs into the brain and the treatment of neurodegenerative diseases (Figure 3).

### 5.5. Involved in Peripheral Nerve Injury

The involvement of radixin in peripheral nerve injury has also been investigated. In response to nerve injury, the increased expression of activated ERM was observed in the spinal microglia [75]. ERM expression has been associated with nerve pain since ERM antisense administration attenuated nerve injury-induced tactile allodynia accompanied by decreased phosphorylation of ERM, suggesting that ERM activation in spinal microglia contributes to nerve injury-induced neuropathic pain [75].

**Figure 4:**
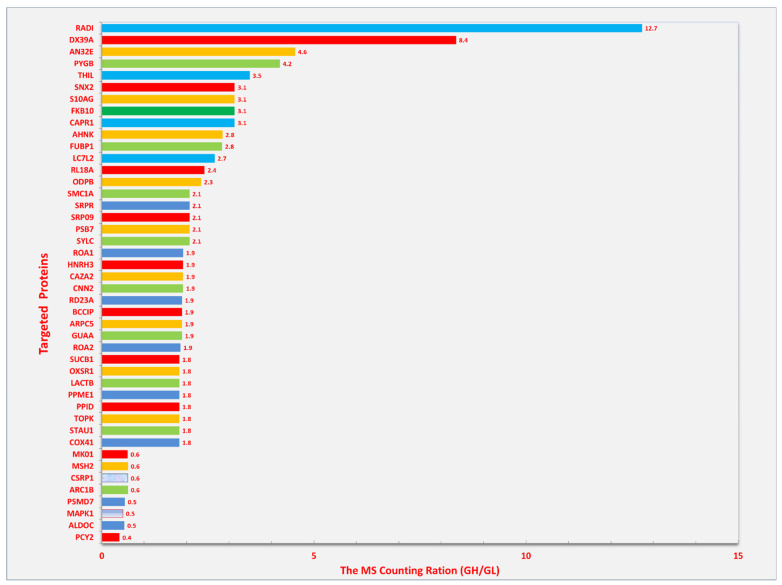
The spectral counting ratio of radixin in Schwann cells after elevated glucose treatment. The Human Schwann cell line was cultured at 37 °C in a humidified atmosphere (5%C02/95% air) in Dulbecco’s modified Eagle’s medium supplemented with 10% fetal bovine serum. When cells were at 50–70% confluence, the culture medium was changed with either low (GL, 5.5 mM) or high (GH, 33 mM) glucose, and the cell culture continued. Then, the cell protein extracts were prepared after the cultures had been maintained under low or high glucose for 72 h. The samples of cells were fully separated on SDS-PAGE and divided into two groups (high abundant protein gel band and low abundant protein gel band). The resulting 4-peptide samples were analyzed by liquid chromatography-mass spectrometry (LC-MS/MS) on Orbitrap Velos MS instrument. The MS/MS spectra were searched against a Swissport human database using a local MASCOT search engine (V.2.3). The relative protein abundance was calculated based on spectrum counting. The significance (*p* < 0.05) of the ratio was achieved by t-test between the low and high glucose groups, *n* = 3. RADI: radixin.

We have found significant changes in radixin in Schwann cells treated with elevated glucose concentrations. Schwann cells are essential for the axonal myelination of the peripheral nerves. We treated cultured Schwann cells with elevated concentrations of glucose. The proteins were run through liquid chromatography-tandem mass spectrometry (LC-MS/MS). The protein abundance was evaluated based on spectrum counting. As shown in Figure 4, several cellular proteins were significantly elevated in spectrum counting after treatment with glucose (33 mM), among which, radixin showed the greatest increase in spectral counting. Considering the association between radixin and nerve injury, our results indicated that radixin might participate in peripheral nerve injury in diabetic neuropathy. Further study should investigate the role of radixin in diabetes-induced peripheral neuropathy *in vivo* and associated mechanisms.

## 6. The Roles of Radixin in Cancer

### 6.1. Cancer Growth

Radixin has been demonstrated to increase the growth, migration, and invasibility of cancer cells. High expression of radixin in glioblastoma U251 cells, prostatic hyperplasia, and neoplasia were observed [76,77]. The suppression of radixin upregulates thrombospondin-1 (TSP-1) and E-cadherin and downregulates matrix metalloproteinase (MMP) -9 in glioblastoma U251 cells, which may be associated with cell migration and invasion [76]. The knockdown of radixin also inhibits the metastasis of human gastric carcinoma cells in vitro by upregulating E-cadherin [78]. These results indicate that radixin might promote the invasibility of glioblastoma and gastric carcinoma. In addition, radixin increases the invasibility of colon cancer cells by increasing the expression of MMP-7 [79]. Radixin gene suppression inhibited cell proliferation, survival, and invasibility of human pancreatic cancer cells. The implantation of radixin-deficient cells in mice also reduced the density of microvessels and inhibited tumor growth [80].

The regulation of integrin-associated protein CD47 by radixin has been linked to poor prognosis of cancers. Radixin has been demonstrated to regulate the localization of CD47. CD47 is a transmembrane protein found on many cancer cells, contributing to the poor prognosis of cancers. Although CD47 was shown to co-localize with ezrin, radixin, and moesin, only radixin gene silencing downregulated the expression of CD47 on the plasma membrane, suggesting that radixin may be the only ERM protein that regulates the CD47 plasma membrane localization [81], and a possible mechanism that radixin promotes the evasion of cancer cells. Similar results were obtained by the same research group in pancreatic ductal adenocarcinoma cells [82].

The expression and function of ERM proteins in cancer cells may not be all the same. The mRNA expression of radixin, moesin, and ezrin was decreased at the early stage bronchioloalveolar carcinomas and in invasive lung adenocarcinomas. Ezrin expression was retained in most tumor cells [83]. Radixin, moesin, and ezrin were preferentially distributed on the plasma membrane in human colon adenocarcinoma cells, However, the mRNA levels of ezrin and moesin were observed to be higher than those of radixin. Although radixin, moesin, and ezrin proteins were all highly colocalized with P-gp, only ezrin seemed to primarily regulate the cell surface distribution and transportation of P-gp [84]. Ezrin and radixin were also demonstrated to regulate the plasma membrane expression of programmed cell death ligand-1 (PD-L1), since ezrin and radixin, but not moesin, gene silencing significantly reduced PD-L1 expression [84].

### 6.2. Drug Resistance

Radixin has been demonstrated to play a role in drug resistance in chemotherapy by upregulating the expression of multidrug resistance-associated protein 2 (MRP2). Radixin knockdown in human cell lines, including liver cancer, lung carcinoma, and breast carcinoma, significantly inhibited MRP2 activity, facilitating the entry of methotrexate into the cells [85]. In human gastric carcinoma cells that express all three ERM proteins, radixin knockdown reduced the expression of both MRP2 mRNA and protein, leading to a decrease of the efflux ability [45]. In human intestinal epithelial cells, loss of either radixin or ezrin independently caused reduced MRP2 expression on the cell surface [86]. The expression of MRP2 and radixin is confined to the membrane of canaliculi in normal hepatocytes; however, cellular irregular MRP2 immunostaining area with colocalized radixin in non-icteric primary biliary cirrhosis was reduced, suggesting that radixin redistribution promotes canalicular MRP2 expression [87]. Increased expression of radixin during epithelial–mesenchymal transition induced by snail family zinc finger 1 (SNAI1) was observed in hepatoblastoma-derived HepG2 cells. The increased radixin enhanced the expression of membrane P-gp, leading to increased drug resistance [88].

## 7. The Role of Radixin in Diabetes Mellitus

As mentioned in Section 5, radixin may play a potential role in diabetic neuropathy. ERM proteins have also been considered new binding proteins of advanced glycation endproducts (AGEs) [89], implicating that ERM proteins may be associated with AGEs in the development of diabetic complications. AGEs were reported to inhibit tubulogenic and migration of kidney epithelial cells in an ezrin-dependent manner [90]. Further studies indicated that glycated proteins could bind to the N-terminal domain of ezrin and inhibit its phosphorylation [91], which seems to facilitate calpain-meditated ezrin cleavage [92]. However, the similar roles of radixin have not yet been reported.

Activation of moesin was reported to suppress AGE-induced angiogenesis. Moesin phosphorylation at Thr558 inhibited AGE-induced proliferation, migration, and tube formation of human umbilical vein endothelial cells (HUVECs). In this process, AGE-induced phosphorylation of moesin was dependent on both RhoA and ROCK, since inhibition of RhoA and ROCK decreased AGE-induced moesin phosphorylation and repressed HUVEC angiogenesis [93]. Moreover, AGEs-induced activation of ROCK phosphorylates moesin, promoting the interaction between moesin with CD44, which might subsequently stimulate the migration of retinal microvascular pericytes (RMPs). The process may push RMP detachment in the microvessel, suggesting that moesin may play a role in retinal detachment in diabetes [94].

## 8. Other Possible Roles of Radixin

Changes in radixin levels have been observed in some diseases other than cancer. In adjuvant-induced arthritis, the radixin mRNA was decreased significantly in the liver without changes in the kidney, small intestine, or the brain. The expression of membrane radixin and activated radixin was also decreased in the liver in adjuvant-induced arthritis [95]. Further study indicates that the reduced radixin leads to the impairment of interaction between radixin and efflux transporters in the liver, reducing the formation of radixin, MRP-2, and P-gp complex [95,96]. Radixin also maintains the canalicular structure and the homeostasis of the canalicular pole in hepatocytes [97]. The expression of radixin was found in canalicular membrane vacuoles, which colocalized with MRP-2 [98]. Radixin has been shown to be essential for maintaining canalicular membrane transporters [99]. The export pump dysfunction at the canalicular membrane of the hepatocyte can result in excretory liver failure; for example, the dysfunction of radixin aggravates cholestasis.

Human ERM proteins have also been shown to differentially regulate hepatitis C virus (HCV) infection. Radixin and moesin expression significantly decreased in HCV-infected patient livers. CD81 is a host receptor for HCV and can phosphorylate ezrin and radixin via activating spleen tyrosine kinase (SYK). Overexpression of moesin or radixin significantly decreased the expression of HCV proteins. In contrast, transient knockdown of moesin or radixin facilitated the infection of HCV [100]. Moreover, a human radixin hinge region peptide (Peptide1) can specifically block the HCV virus entry in Huh7.5 cells [101].

## 9. Targeting Radixin for Therapeutic Applications

In the above sections, the involvement of radixin and other ERMs in some diseases has been discussed. Therefore, targeting radixin may hold potential for novel treatment strategies for these diseases.

### 9.1. Neurodegenerative Diseases

In neurodegenerative diseases, such as Alzheimer’s disease (AD), moesin has been associated with cognitive decline. Moesin was found to be a highly abundant protein in plaque-associated microglia in human AD. Moesin is positively associated with β-amyloid plaques, neurofibrillary tangles, and cognitive dysfunction [102]. The FERM domain of moesin and radixin can interact with CD44, while some compounds that inhibit the interaction between moesin and CD44 have been shown to attenuate AD-associated neuronal injury. These results suggest that the FERM domain holds potential as a drug development target for AD [103].

### 9.2. Cancers

Targeting radixin may function to inhibit gastric cancer tumorigenicity and metastasis. In gastric cancer tissues, the decreasing expression of intercellular adhesion molecule 2 (ICAM2) was found to correlate with advanced stages and metastasis in cancer patients positively. In gastric cancer cells, ICAM2 decreased the expression of radixin. Further study indicates that ICAM2 binds to radixin to promote the ubiquitination and degradation of radixin. The results implicate that reducing the expression of radixin through modulators possesses possible therapeutic effects on gastric cancer [104].

Similarly, higher moesin expression has been associated with poor prognosis in patients with colorectal cancer. The mechanism study illustrated that moesin regulates the expression of runt-related transcription factor 2 (RUNX2) by activating β-catenin signaling. Overexpression of moesin promotes cancer cells to proliferate and migrate via the β-catenin-RUNX2 interaction, suggesting that moesin may function as a potential therapeutic target for colorectal cancer [105].

In cancers, ERM proteins are also associated with programmed cell death-1 (PD-1). Programmed cell death ligand-1 (PD-L1) binds to PD-1 to deregulate T-cell function and promote tumor aggressiveness. ERM proteins were illustrated to colocalize with PD-L1 in the plasma membrane and interact with PD-L1 in colorectal cancer cells. Gene silencing of ezrin and radixin reduced the expression of PD-L1 on the cell surface [84]. The results implicate that targeting radixin and ezrin may facilitate finding novel treatments for colorectal and other types of cancers.

Interestingly, the expression of moesin was downregulated in breast cancers. ERM proteins can interact with HER2 to control the localization of HER2 and prevent HER2 activation. In HER2-positive breast cancers, moesin expression decreased, which correlated with increased HER2 expression. Manipulating the increase of the expression of ERM proteins in HER2-positive breast cancer cells inhibited HER2 activation, resulting in the repression of HER2-dependent cell proliferation [106]. The role of moesin in keeping HER2 in a repressed status may provide novel treatment approaches by targeting moesin for HER2-positive breast cancer.

Moreover, pharmacological inhibition of ERM activation showed growth suppression of rhabdomyosarcoma. Application of a small molecule pharmacophore NSC668394 to inhibit ERM phosphorylation in rhabdomyosarcoma cells decreased the cell viability and proliferation in a dose-dependent manner. The underlying mechanism may be related to the induction of caspase-3 activation and apoptosis.

The studies mentioned above demonstrate that targeting ERM proteins for inhibition can repress the growth of cancer cells and prevent cancer cell invasion, possibly improving the prognosis of cancer patients. However, the studies are preliminary and remain to be elaborated upon to be practical in clinical settings. Either pharmacological inhibition or molecular silencing of ERM genes has been tried, but these are insufficient to conclude the therapeutic capability of targeting ERM proteins for cancers. Extensive studies will be required to further refine the therapeutic application by modulating the ERMs. More practical pharmacological drugs and molecular reagents need to be developed, and large clinical trials will be required to test the efficacy of ERM modulators in cancers.

## 10. Conclusions

Radixin, as a scaffolding protein, plays important roles in the nervous system and other body systems. Radixin primarily interacts with multiple membrane proteins and is activated by phosphorylation. Radixin has been shown to promote axonal growth and prevent neurodegeneration. Its aberrant expression is involved in the pathogenic process of diseases in the central nervous system and peripheral nerve injury. In addition, radixin improves the growth of cancer cells, increases drug resistance for chemotherapy, and is involved in the development of diabetic complications. Understanding the mechanisms by which radixin mediates the cell signaling pathways for its biological activities may hold a great opportunity to find therapeutic strategies for diseases in the nervous system, cancers, and other diseases.

## Figures and Tables

**Table 1:** Phosphorylation of ERM-associated kinases and bioactivity.

Phosphorylated Residues	Kinases	Function	References
Thr567 of Ezrin	GRK2	G protein-coupled receptor-dependent reorganization of the actin cytoskeleton. Regulates membrane protrusion and motility in epithelial cells.	[11,12]
	NIK	Lamellipodium extension induced by growth factors.	[13]
	LOK	Regulates cytoskeletal rearrangement of lymphocytes.	[14]
	PKC	Regulates osteosarcoma cell migration. Regulates endothelial permeability.	[19,20]
Thr235 of Ezrin	CDK5	Mediates pRb-induced cell shape changes in senescent cells.	[21,22]
Thr558 of Moesin	RhoK	Mediates the formation of microvilli-like structures. Mediates glutamate-induced phosphorylation in neurons and post-injury regeneration of neurons.	[23,24,25]
	Akt	Mediates neurite formation in vitro.	[26]
Thr567 of Ezrin Thr558 of Moesin	ROCK	Involved in the early steps of apoptotic signaling following Fas triggering and regulates apoptosis induction.	[27]
	Cdc42	Involved in the formation of filopodia.	[28]
	PKC	Regulates endothelial permeability.	[20]
Thr564 of Radixin	Akt	Involves neurite outgrowth and growth cone formation.	[9]
	GRK2	Regulates membrane protrusion and motility in epithelial cells.	[12]
	PKC	Regulates endothelial permeability.	[20]
Thr573 of Radixin	Akt	Stabilizes radixin interactions with F-actin to regulate neurite outgrowth. Inhibits ubiquitin-dependent proteasomal degradation of radixin.	[9]
Thr567 of Ezrin Thr558 of Moesin Thr564 of Radixin	LRRK2	Involved in neuronal growth cone development.	[29,30]
Tyr145, 353, and 477 of Ezrin	SK ITK	Phosphorylation of Tyr145 and 477 involves cell adhesion and migration, whereas Tyr353 phosphorylation regulates the reorganization of the actin cytoskeleton and activation of B cells.	[31,32,33]

Note: Cdc4, cell division control protein 4; CDK5, cyclic-dependent kinase 5; GRK2, G-protein coupled receptor kinase 2; ITK, intrinsic Tyr kinase; LOK, lymphocyte-oriented-kinase; LRRK2, leucine-rich repeat kinase-2; NIK, nick interacting kinase; PKC, protein kinase C; ROCK, rho-associated coiled coil-containing protein kinase; RhoK, rho-associated kinase; SK, Src kinases.

## Data Availability

Not applicable.
